# The application of augmented reality in craniofacial bone fracture reduction: study protocol for a randomized controlled trial

**DOI:** 10.1186/s13063-022-06174-3

**Published:** 2022-03-29

**Authors:** Li Lin, Xiangqi Liu, Yuan Gao, Zin Mar Aung, Haisong Xu, Bingshun Wang, Le Xie, Xianxian Yang, Gang Chai

**Affiliations:** 1 Department of Plastic and Reconstructive Surgery, Shanghai 9th People’s Hospital, School of Medicine, Shanghai Jiao Tong University, 639 Zhi Zao Ju Rd, Shanghai, 200011 China; 2 Institute of Forming Technology & Equipment, Shanghai Jiao Tong University, Xuhui Campus, 1954 Hua Shan Rd, Shanghai, 200030 China; 3 Department of Biostatistics, Clinical Research Institute, Shanghai Jiao Tong University School of Medicine, 227 Chong Qing Nan Rd, Shanghai, 200025 China; 4 National Digital Manufacturing Technology Center, Shanghai Jiao Tong University, Xuhui Campus, 1954 Hua Shan Rd, Shanghai, 200030 China; 5 Institute of Medical Robotics, Shanghai Jiao Tong University, Minhang Campus, 800 Dong Chuan Rd, Shanghai, 200240 China; 6 Department of Plastic and Reconstructive Surgery, Maternal and Child Health Care Hospital of Hainan Province, Haikou, 570206 China

**Keywords:** Augmented reality, Zygomaticomaxillary complex fractures, Reduction surgery

## Abstract

**Background:**

Augmented reality (AR) is a new technology that increases users’ perception of the real world. The purpose of this study is to evaluate the efficacy and safety of augmented reality navigation system in treatment with craniofacial fracture reduction.

**Methods:**

This will be a single-center prospective randomized controlled trial. Twenty-two patients will be assigned to two groups of 11, and those with zygomaticomaxillary complex fractures will undergo preoperative three-dimensional CT modeling and have operational plans designed. The control team will use traditional optical navigation to perform the surgery, and the experimental team will use an AR navigation system. The primary outcome measures will be the accuracy of the key points of surgical area between the preoperational surgical plan and post-operation. The secondary outcome measures will be the blood loss, operation time, bone reduction time, hospital time, and complication rate. The findings obtained through this study are expected to evaluate efficacy and safety of the augmented reality navigation system in the treatment of zygomaticomaxillary complex fractures.

**Discussion:**

This controlled trial of augmented reality navigation system in treatment with zygomaticomaxillary complex fracture reduction will clarify the efficacy and safety of this technology by measuring the accuracy of the key points of surgical area and blood loss, operation and bone reduction times, hospital stay duration, and complication rates. This is a single-center study, and the results are expected to promote the application of augmented reality in craniofacial fracture reduction to improve surgery accuracy and efficacy.

**Trial registration:**

Chinese Clinical Trial Registry ChiCTR1900022626. Registered on April 19, 2019.

## Administrative information

**Table:** 

Title {1}	The application of augmented reality in craniofacial bone fracture reduction: Study protocol for a randomized controlled trial
Trial registration {2a and 2b}.	ChiCTR1900022626. (https://www.chictr.org.cn/showproj.aspx?proj=38142) Registered on April 19, 2019.
Protocol version {3}	April 19, 2019 Original version
Funding {4}	This work was supported by the project of Science and Technology Commission of Shanghai Municipality (No. 19441912300); Clinical Research Plan of SHDC (No. SHDC2020CR3070B); The project of Shanghai Jiao Tong University School of Medicine Two-hundred Talent (No. 20161420) and the project of Shanghai Municipal Key Clinical Specialty (shslczdzk00901).
Author details {5a}	^1^Department of Plastic and Reconstructive Surgery, Shanghai 9th People's Hospital, School of Medicine, Shanghai Jiao Tong University, 639 Zhi Zao Ju Rd, Shanghai, 200011, China ^2^Institute of Forming Technology & Equipment, Shanghai Jiao Tong University, Xuhui Campus, 1954 Hua Shan Rd, Shanghai, 200030, China ^3^Institute of Medical Robotics, Shanghai Jiao Tong University, Minhang Campus, 800 Dong Chuan Rd, Shanghai, 200240, China ^4^Department of Plastic and Reconstructive Surgery, Maternal and Child Health Care Hospital of Hainan Province, Haikou, 570206, China ^5^National Digital Manufacturing Technology Center, Shanghai Jiao Tong University, Xuhui Campus, 1954 Hua Shan Rd, Shanghai, 200030, China ^6^Department of Biostatistics, Clinical Research Institute, Shanghai Jiao Tong University School of Medicine, 227 Chong Qing Nan Rd, Shanghai 200025, China
Name and contact information for the trial sponsor {5b}	Gang Chai, Department of Plastic and Reconstructive Surgery, Shanghai 9th People's Hospital, School of Medicine, Shanghai Jiao Tong University, 639 Zhi Zao Ju Rd, Shanghai, 200011, China Xianxian Yang, Department of Plastic and Reconstructive Surgery, Shanghai 9th People's Hospital, School of Medicine, Shanghai Jiao Tong University, 639 Zhi Zao Ju Road, Shanghai, 200011, China
Role of sponsor {5c}	The funding agency did not take part in the study design, or data collection or any other part of the trial. The sponsor declares that there is no commercial interest in the trial.

## Introduction

### Background and rationale {6a}

The zygomaticomaxillary complex (ZMC) is an integral part of the facial skeleton. There are four articulations in the anatomy of ZMC including the zygomaticosphenoid suture, zygomatic arch with an articulation with the temporal bone, zygomaticomaxillary buttress, and the zygomaticofrontal suture. A bony fracture of the ZMC can damage one or more of the four articulations causing functional and cosmetic problems [1–5].

In traditional ZMC fracture reduction surgery, surgeons usually conduct the preoperative surgical simulation mentally to determine the correct operational procedure and ensure the success of the operation. As a result, the quality of the surgical plan often depends on the surgeon’s own clinical experience and surgical skill, and it is difficult for every member of the surgical group to share that information about the surgical plan [6–10].

Several techniques are used for accurate ZMC fracture reduction, but there are still many problems needed to be solved such as surgical navigation, virtual surgery planning, and a three-dimensional (3D) printing template [11–13]. Augmented reality (AR) is a new technology that increases users' perception of the real world through real and virtual information provided by computer systems. The three characteristics include combining the real and virtual, real time inter-reactivity, and registration in 3D [14].The emergence of AR technology, which uses computers instead of surgeons to carry out the 3D conception of surgical plans, is objective and quantifiable and can be shared by other members of the surgical community.

Recent reviews indicate that AR could provide benefits that address the challenges of conventional navigation systems, such as hand-eye coordination and depth perception [15–19]. The AR study was started in craniofacial surgery. Similar studies have demonstrated the efficacy of the AR system in treating hemifacial microsomia [20], orbital hypertelorism [21], craniosynostosis [22], and craniofacial contouring surgery [23]. While there was a study on the application of AR to the treatment of unilateral orbitozygomatic maxillary complex fracture [24], it was a single-arm study with small sample size. Thus, the purpose of this randomized controlled study is to evaluate the efficacy and safety of AR system in treatment with craniofacial fracture reduction.

### Objectives {7}

The purpose of this study is to (1) achieve real-time tracking and dynamic display using an optical marker plate based on the previous developed augmented reality system [25] and (2) evaluate the application effect of this system in ZMC fracture reduction. Through the elaboration of the research methods and results of this topic, this study may provide surgeons with more intuitive medical information and operable method guidance to help surgeons improve the safety and success rate of ZMC surgery.

### Trial design {8}

**Fig. 1:**
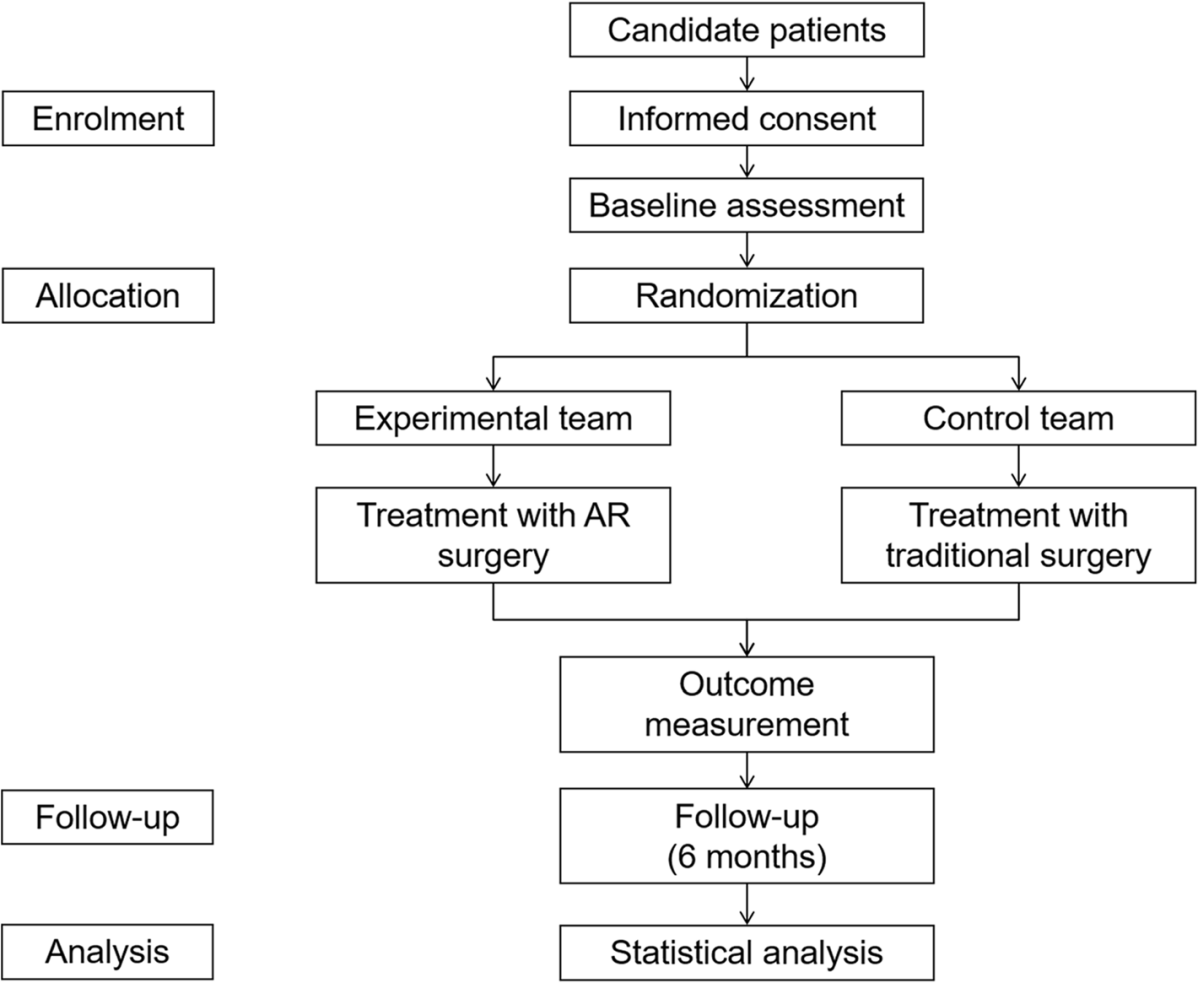
The trial framework. AR, augmented reality

This is a single-center prospective randomized controlled trial, performed in an academic hospital. The two parallel groups in this study include the experimental group patients receiving the AR assistant surgery and the control group to be treated with traditional navigation surgery. The allocation ratio of the two parallel groups is 1:1. The trial framework can be seen in Fig. 1.

## Methods: participants, interventions, and outcomes

### Study setting {9}

Patients with ZMC fracture reduction included in this study are from Shanghai 9th People’s Hospital, School of Medicine, Shanghai Jiao Tong University, which is an academic hospital. The protocol has been registered in the Chinese Clinical Trial Registry center (ChiCTR) with number of ChiCTR1900022626. (https://www.chictr.org.cn/showproj.aspx?proj=38142)

### Eligibility criteria {10}

The inclusion criteria will include (1) preoperative and postoperative three-dimensional head computerized tomography (CT) scans, (2) unilateral zygomaticomaxillary complex fractures of less than 1 month history with no other surgical treatment, (3) no serious underlying diseases or contraindications, (4) no sex restriction with ages from 15- to 65-years-old, and (5) signed informed consent.

The exclusion criteria include (1) compression fractures, fractures older than one month, (2) complex repair surgery requirement, (3) concurrent disease with contraindications for general anesthesia, and (4) no informed consent.

The withdrawal criteria will include (1) failure to receive treatment as planned, (2) patient request to withdraw from the clinical study, and (3) those lost to follow-up.

### Who will take informed consent? {26a}

The trained investigator in the surgical team will introduce the trial to the patients who meet the inclusion criteria and obtain the written consent from the patients that agree to participate.

### Additional consent provisions for collection and use of participant data and biological specimens {26b}

Not applicable as no biological specimens would be collected.

### Interventions

#### Explanation for the choice of comparators {6b}

The control group is treated without the augmented reality for the surgical navigation during the reduction surgery which means that the surgical plan is decided mentally by the surgeon and only optical tracking is used in the operation procedure. This is the traditional treatment for craniofacial reduction surgery.

#### Intervention description {11a}

**Fig. 2:**
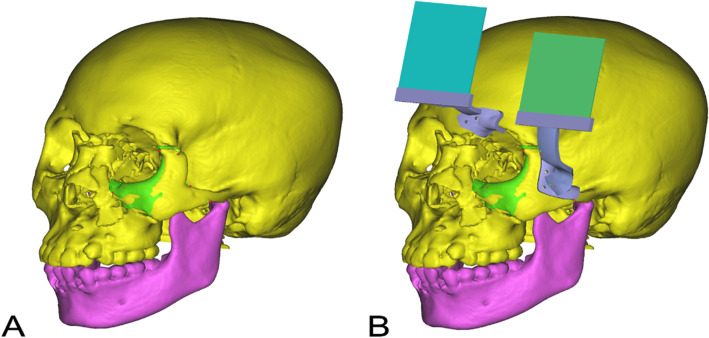
Preoperational surgical design (intervention group). **A** Key points for the fracture bone (red point) and the expected reduction location (green point). **B** Tracking plates for the preoperational design (blue and green rectangles)

**Fig. 3:**
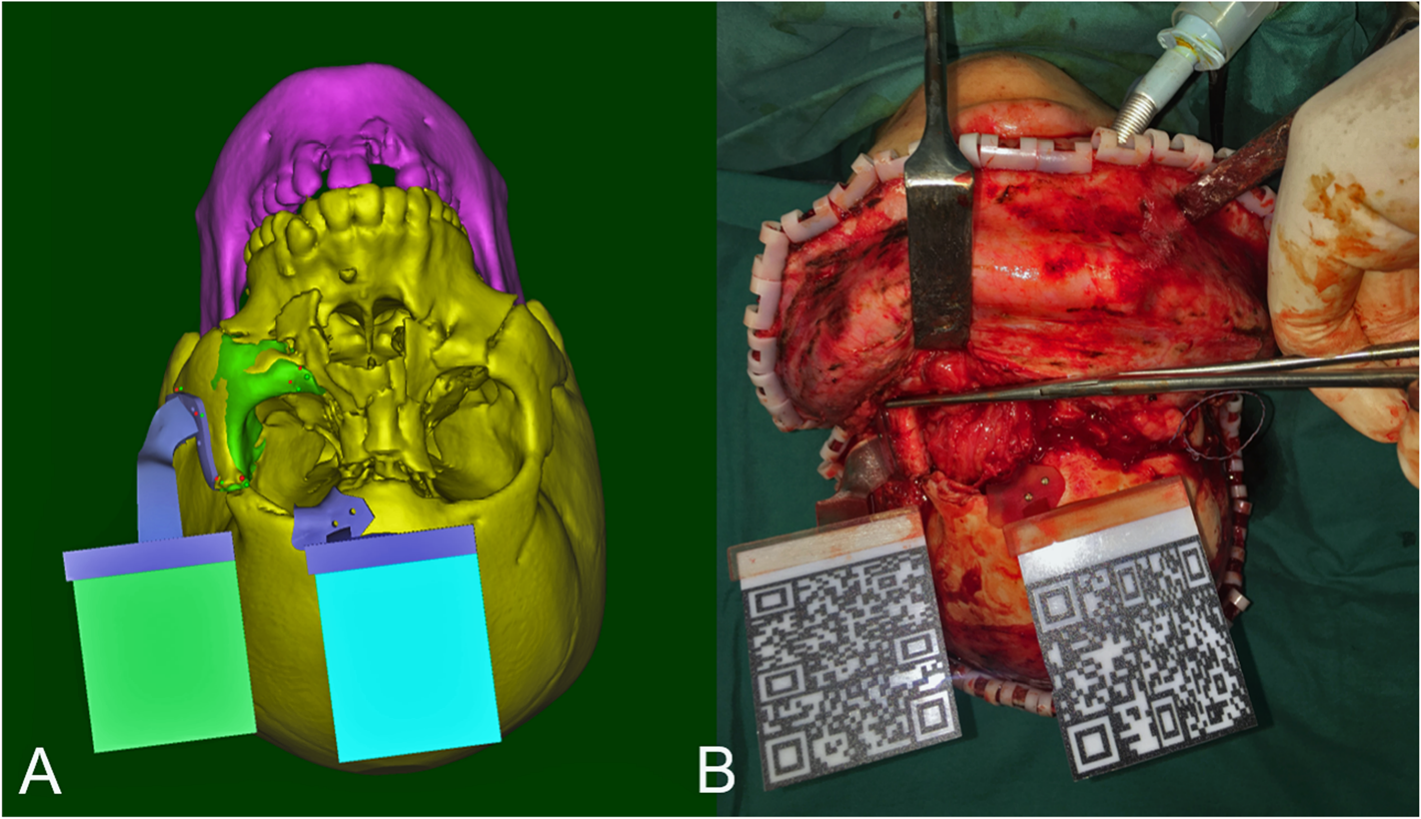
Preoperational design and the matched augmented reality in surgery by 3D printing plates. **A** Preoperational surgical design. **B** Three-dimensional printing tracking plate in the operation for real-time augmented reality tracking system

All patients will undergo a 3D CT scan before surgery (LightSpeed 16, GE, Milwaukee), and the data will be stored in DICOM format. All the digital material will be imported into the corresponding processing software (Mimics 21.0, Materialise, Belgium). In the intervention group, two marker plates will be designed based on the coronal incision that can be fixed on the corresponding bone structure with fixation nails to simultaneously track the fracture block and the whole craniofacial bone (Fig. 2). To ensure the real-time tracking of the markers by the camera, the two-sign plate complexes will be positioned in a parallel arrangement two cm away from the bone surface and oriented as parallel as possible to the coronal surface of the skull (Fig. 3). All marker complexes are obtained using 3D printing (ProJet 660 Pro, 3D Systems, South Carolina) and sterilized before surgery. HoloLens was selected as the hardware support (HoloLens, Microsoft, Washington), and the selected development platform was Unity, written in C# language (Unity, Microsoft, Washington). Tracking will be performed through the Vuforia SDK, which stores the feature points of the logo board in the Vuforia engine database in advance. The video camera of HoloLens is used to track the environment, and the system will continually extract original features from the environment and match them with the template in the database. When matching is successful, the system triggers the display of the model in the set position, which is fixed with the position of the marker board, so the tracking frame rate was guaranteed.

The control team will use traditional ZMC fracture reduction surgery.

#### Criteria for discontinuing or modifying allocated interventions {11b}

The criteria for discontinuing or modifying include the malfunction of AR system, surgical accident, and any situation that make the surgeon need to finish the surgery as soon as possible. The demographic data of the patients who meet those criteria will still be collected.

#### Strategies to improve adherence to interventions {11c}

All the members of the trial team hold Good Clinical Practice (GCP) license from China Food and Drug Administration (CFDA). The Clinical Research Coordinator (CRC) is specialized to inform patients, to follow-up and collect relevant material. There is a statistician to assist the clinical data, which will be collected over 6 months for each patient to follow-up for complications. The principal investigator (PI) will check the process monthly and monitor trial progress. Every quarter, the Clinical Research Center/Office of Quality Management of Shanghai 9th People’s Hospital, School of Medicine, Shanghai Jiao Tong University, will conduct a data audit and verification of the program.

#### Relevant concomitant care permitted or prohibited during the trial {11d}

There is no specific concomitant care or interventions prohibited during the trial. All the patients of the two groups will receive standard perioperative care for their craniofacial fracture.

#### Provisions for post-trial care {30}

The whole surgical team will provide standard postoperative medical care for the participating patients after the trial.

### Outcomes {12}

#### Primary outcome measures

The primary outcome measures will be the accuracy of the key points of surgical areas between preoperational surgical plan and post-operation.

**Fig. 4:**
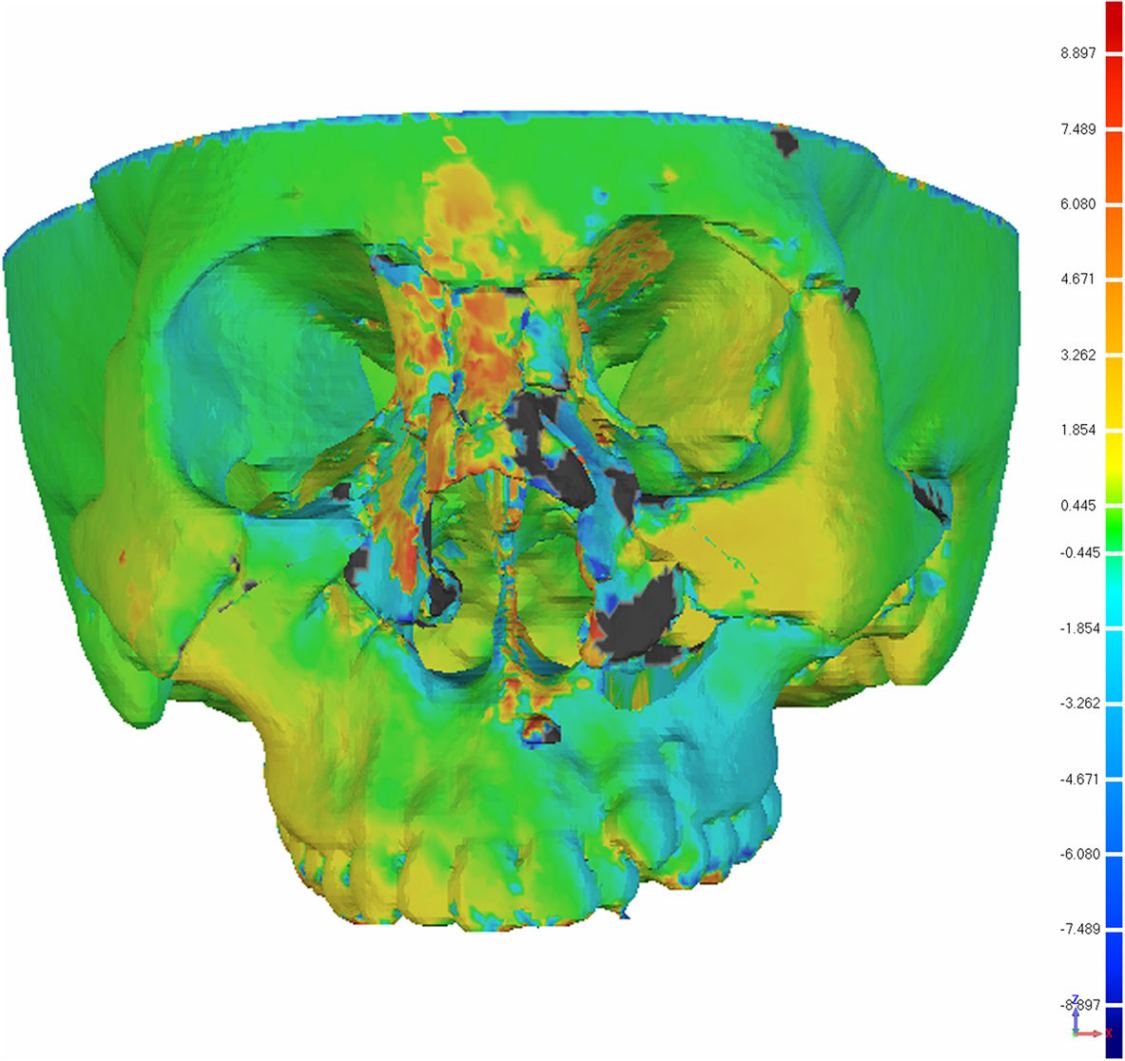
Accuracy of the key points between preoperational surgical plan and 1-week post-operation 3D CT results. The green indicates the most matched area, and the dark blue and red presents the mismatched area

All patients will undergo 3D CT examination 1 week after surgery. The preoperative and postoperative image data will be imported into 3D processing software (Geomagic Control X, 3DSystem, South Carolina). The preoperative and postoperative images will be registered, and the accuracy of the selected fracture points will be measured (Fig. 4). To avoid subjective bias error in measurement, the measurement of this part will be made by the trained engineers who do not participate in the operation and are blinded about the allocation. The measurement will be repeated three times and the average value will be taken as the final data.

#### Second outcome measures

The secondary outcome measures will be blood loss, operation time, bone reduction time, hospital time, and rate of complications. The perioperative period outcome will be obtained from medical records. Any complications will be evaluated during the follow-up period (Table 1).

**Table 1:** The participant and data collection timeline

	Baseline	Allocation	Operation day	Post-operation	
Timepoint	− Day 7	Day 0	Day 1	7 days	6 months
**Enrolment:**					
**Eligibility screen**	X				
**Informed consent**	X				
**Allocation**		X			
**Interventions:**					
**AR surgery**			X		
**Traditional surgery**			X		
**Assessments:**					
**Accuracy**				X	
**Blood loss**			X		
**Operation time**			X		
**Bone reduction time**			X		
**Hospital time**				X	
**Complications**				X	X
**Adverse event**			X	X	X

#### Primary endpoint

The primary endpoint is the success of post-operative 3D CT examination within seven days. The DICOM data will be transferred to the third-party measuring personnel, and all accuracy outcomes will be evaluated in the three-dimensional software.

#### Second endpoint

The second endpoint will be the success follow-up 6 months after operation. The complications during the follow-up period will be summarized as facial asymmetry, scarring, infection, epistaxis, hardware failure, neurapraxia, facial nerve palsy, temperature sensitivity, blindness, decreased visual acuity, diplopia, lid malposition, ectropion or entropion, corneal exposure or abrasion, enophthalmos, epiphora, and orbital dystopia [6]. These complications will be carefully evaluated by surgeons according to the physical examinations table.

### Participant timeline {13}

The participant timeline is shown in Table 1.

### Sample size {14}

This experiment is a single-center prospective randomized controlled trial. Because this kind of experiment has not been carried out previously, the calculated number of samples mainly refers to the previously related published literature [20–24]. With the approval of the ethics committee, the number of patients in each group needs to be 10 for the primary outcome to be statistically significant; meaning a total of 20 patients is needed. The estimated primary outcome of experiment team is 1.5 to 1.6 ± 0.3 mm, while the controlled team value is 2 ± 0.3 mm according to our previous clinical experience. Given the sample size with 10 patients in each group, it achieves 80.491% power to reject the null hypothesis of equal means when the population mean difference is 0.4 with a standard deviation for both groups of 0.3 and with a significance level (α) of 0.050 using a two-sided two-sample equal-variance *t*-test. This sample size achieves 94.081% power to reject the null hypothesis of equal means when the population mean difference is 0.5 with a standard deviation for both groups of 0.3 and with a significance level (α) of 0.050 using a two-sided two-sample equal-variance *t*-test. To allow for a 10% potential dropout rate, the target sample size of this study will be set to 22.

### Recruitment {15}

This trial is going to be performed in Shanghai 9th People’s Hospital, School of Medicine, Shanghai Jiao Tong University, which is an academic hospital. This hospital is well-known for its plastic and reconstruction surgery and there is a great amount of craniofacial fracture patients having surgery annually which guarantees the targeted number of participants will be enrolled.

### Assignment of interventions: allocation

#### Sequence generation {16a}

In this randomized controlled study, **t**he permuted block randomized assignments list (1:1) has been used for grouping. One group is designated the control team and the other group was designated the experimental team. The random distribution list is developed and provided by a research statistician.

#### Concealment mechanism {16b}

All the parameters of the random distribution list and the random list itself were considered part of the blinded background and were stored in sealed envelopes. Before the blind is uncovered, all patients participating in the trial are kept confidential. Specific ID numbers are adopted. The identifiable information and study data is separated to ensure that the measurers are blind.

During the trial, the operators are unable to design the blind method because he knew which way to use the intervention. Neither the enrolled patients nor the data measurer knew which group the patients were assigned to as part of a single blind trial.

#### Implementation {16c}

First, the investigators in the trial team will take responsibility of enrolling the participants who fulfill the inclusion criteria and get the consent. Then, the research statisticians conduct the randomized allocation and sent the randomized number of the patient. The surgeons will perform the surgery for the patient with or without the AR system according to the allocation. And the engineer who is blinded about the allocation measure the primary outcome.

### Assignment of interventions: blinding

#### Who will be blinded {17a}

The operator is unable to design the blind method because he knew which way to use the intervention. The enrolled patients and the data measurer do not know whether they are undergoing surgery in the augmented reality assisted mode and conducted a single blind trial.

#### Procedure for unblinding if needed {17b}

This is a single blind trial, only the patients and measurer are blinded, and there is no circumstance that they need to be unblinded.

### Data collection and management

#### Plans for assessment and collection of outcomes {18a}

The preoperative and postoperative image data will be imported into 3D processing software (Geomagic Control X, 3DSystem, South Carolina). The pre- and postoperative images will be registered, and the accuracy of the selected fracture points was measured.

The primary outcome measures will be the accuracy of the key points of surgical site between the preoperational surgical plan and post-operation outcome. The post operational CT will be within 7 days after surgery, and this is the key outcome for the effectiveness of AR technology.

For security measures including intraoperative blood loss, operation time, bone reduction time, hospital time, possible blood transfusion, and rates of complications, it will be arranged for surgeons to record relevant information after surgery within the follow-up period.

#### Plans to promote participant retention and complete follow-up {18b}

Patients with craniofacial bone fracture care about the prognosis for it not only affects the function but also the esthetic issue. Besides, it is routine to record the patients’ contact information such as mobile phone number, the ID of Wechat, and the email. The patients also have the phone number of our center to contact our medical provider for any questions. In this way, we keep in close touch with the patients to insure the follow-up. In addition, some of the patients need to come back to our hospital for other treatment of combined injuries, like the ophthalmic issue. The medical team will provide standard pre- and post-operative care for trial patients to promote their retention for free. All patients will have 6 months follow-up for postoperative recovery and monitoring for complications and the PI will check the process and listen to the report of the investigator. Every quarter, the Clinical Research Center/Office of Quality Management of Shanghai 9th People's Hospital, School of Medicine, Shanghai Jiao Tong University, will conduct a data audit and verification of the program.

#### Data management {19}

The preoperative and postoperative image data will be imported into 3D processing software (Geomagic Control X, 3DSystem, South Carolina). The pre- and postoperative images will be registered and the accuracy of the selected fracture points measured.

The primary outcome measures will be the accuracy of the key points of surgical site between the preoperational surgical plan and post-operation outcome. The post operational CT will be within 7 days after surgery, as the key outcome for the effectivity of AR technology.

For security measures including intraoperative blood loss, operation time, bone reduction time, hospital time, possible blood transfusion, and rates of complications, this team will arrange surgeons to record relevant information after surgery within the follow-up period.

#### Confidentiality {27}

The personal information of all the participants will be collected by trained investigators and stored in specific cabinets with limited access. All the digital data in the measurement software will be accessible only by password for the staff in charge of measuring the pre- and postoperative 3D data.

#### Plans for collection, laboratory evaluation, and storage of biological specimens for genetic or molecular analysis in this trial/future use {33}

Not applicable. No biological specimen is needed in this trial.

### Statistical methods

#### Statistical methods for primary and secondary outcomes {20a}

Primary and secondary outcomes will be analyzed and processed with SAS software, version 9.4 (SAS Institute, USA). All measurement data will be expressed as *N* (%) and mean (± standard deviation) with median and interquartile range when appropriate. Comparisons between two groups will be made by Student’s *t*-test when the data was normally distributed. Fisher’s exact test will be used for the comparison of enumeration data between groups as the sample size was less than 40. *P* < 0.05 will be considered statistically significant.

### Interim analyses {21b}

Not applicable. The study period is short, and no serious potential adverse event would occur.

#### Methods for additional analyses (e.g., subgroup analyses) {20b}

Not applicable. There is no subgroup analysis.

#### Methods in analysis to handle protocol non-adherence and any statistical methods to handle missing data {20c}

In this trial, the analysis will be performed based on the intention to treat (ITT) principle. The ITT will also be used to deal with those who meet withdrawal criteria, including their demographic data. In case of missing data, the statistician will adopt the last observation carried forward (LOCF) to complete the analysis.

#### Plans to give access to the full protocol, participant level-data and statistical code {31c}

The full protocol can be accessed by the website of https://www.chictr.org.cn/showproj.aspx?proj=38142. The data of the key findings and statistical code will be available on trial publication.

### Oversight and monitoring

#### Composition of the coordinating center and trial steering committee {5d}

This is a single-center trial without coordinating center. The trial staff consists of trained investigators taking responsibility for enrolment, clinical data collection, digital data measurement, and the surgical team providing the procedure and medical care. The PI of this trial will check the process and listen to the report of the trial from those investigators every month. The trial steering committee providing oversight of this trial is the Clinical Research Center/Office of Quality Management of Shanghai 9th People’s Hospital, School of Medicine, Shanghai Jiao Tong University, who will review the data and check the research progress every quarter.

#### Composition of the data monitoring committee, its role and reporting structure {21a}

The Clinical Research Center/Office of Quality Management of Shanghai 9th People’s Hospital, School of Medicine, Shanghai Jiao Tong University, is the data monitoring committee of this trial; the staff and experts of the office will perform the audit and review the research data every quarter. It is independent from the sponsor and has no competing interests.

#### Adverse event reporting and harms {22}

According to the published literature on the application of AR in other kinds of surgeries, there is no reported adverse event related to this technique. The clinical doctor in the team will record and provide medical care if any complications occur in either group within the follow-up duration and report it to the ethical committee in a week.

#### Frequency and plans for auditing trial conduct {23}

Every month, the PI will check the process and listen to the report of this trial. Every quarter, the Clinical Research Center/Office of Quality Management of Shanghai 9th People’s Hospital, School of Medicine, Shanghai Jiao Tong University, will conduct data audit and verification of program.

#### Plans for communicating important protocol amendments to relevant parties (e.g. trial participants, ethical committees) {25}

Any amendments of the protocol will be submitted to the ethical committee and implemented once approved.

#### Dissemination plans {31a}

The findings of this study will be disseminated through peer-reviewed publications and conference presentations and the privacy and interest of the patients will be protected. Data that would break the blind rules will not be released before completion of the trial. The PI, Dr. Gang Chai, will take the lead in publication and will be acknowledged in publications.

## Discussion

This article presents a randomized controlled trial that aims to examine the efficacy of AR navigation for the treatment of ZMC fractures. The ZMC is a complicated anatomy containing four articulations, so the reduction surgery needs higher accuracy and stability to achieve satisfying functional and cosmetic outcomes. Augmented reality technology is a new computer-assisted surgery system based on virtual reality, featuring the combination of the real and virtual world and providing real-time navigation for the surgeon based on the 3D model. Current literature proves that it benefits the hand-eye coordination and depth perception in various kinds of surgeries. The results of this trial are expected to promote the application of AR technology in ZMC fracture surgery. Based on previous experimental studies on the application of AR technology in other kinds of surgeries, the sample size of this trial is small; therefore, the further step would be a larger sample size and multicenter trial. This trial will provide important data for the definitive trial design to gain clinical value and sustained improvements in the use of AR technology in ZMC fracture reduction.

## Trial status

Protocol version = 1

Date of register = April 19, 2019

Date recruitment began = April 18, 2019

Estimate completion date = May 2022 (for the epidemic of COVID-19, the estimated completion date has been extended)

## Abbreviations

AR: Augmented reality
ZMC: Zygomaticomaxillary complex
VR: Virtual reality
PI: Principle investigator
3D: Three-dimensional
ITT: Intention to treat
LOCF: Last observation carried forward
GCP: Good Clinical Practice
CFDA: China Food and Drug Administration
CRC: Clinical Research Coordinator
